# A Multicentre Study of Acute Kidney Injury in Severe Sepsis and Septic Shock: Association with Inflammatory Phenotype and HLA Genotype

**DOI:** 10.1371/journal.pone.0035838

**Published:** 2012-06-06

**Authors:** Didier Payen, Anne-Claire Lukaszewicz, Matthieu Legrand, Etienne Gayat, Valérie Faivre, Bruno Megarbane, Elie Azoulay, Fabienne Fieux, Dominique Charron, Pascale Loiseau, Marc Busson

**Affiliations:** 1 Department of Anesthesiology and Critical Care, Lariboisière Hospital, Assistance Publique-Hôpitaux de Paris, Paris, France; 2 Medical Intensive Care Unit, Lariboisière Hospital, Assistance Publique-Hôpitaux de Paris, Paris, France; 3 Medical Intensive Care Unit, Assistance Publique-Hopitaux de Paris, Paris, France; 4 Surgical Intensive Care Unit, Assistance Publique-Hopitaux de Paris, Paris, France; 5 INSERM UMR 940 Saint-Louis Hospital, Assistance Publique-Hopitaux de Paris, Paris, France; 6 University Paris 7 Denis Diderot, Sorbonne Paris Cité, Paris, France; University of Sao Paulo Medical School, Brazil

## Abstract

**Background:**

To investigate the association between severity of acute kidney injury (AKI) and outcome, systemic inflammatory phenotype and HLA genotype in severe sepsis.

**Methodology/Principal Findings:**

Prospective multicenter observational study done in 4 intensive care units in two university hospitals**.** Severe sepsis and septic shock patients with at least 2 organ failures based on the SOFA score were classified: 1) "no AKI", 2) "mild AKI" (grouping stage 1 and 2 of AKIN score) and 3) "severe AKI" (stage 3 of AKIN score). Sequential measurements**:** The vasopressor dependency index (VDI; dose and types of drugs) to evaluate the association between hemodynamic status and the development of early AKI; plasma levels of IL-10, macrophage migration inhibitory factor (MIF), IL-6 and HLA-DR monocyte expression. Genotyping of the 13 HLA-DRB1 alleles with deduction of presence of HLA-DRB3, -DRB4 and -DRB5 genes. We used multivariate analysis with competitive risk model to study associations. Overall, 176 study patients (146 with septic shock) were classified from AKIN score as "no AKI" (n = 43), "mild AKI" (n = 74) or "severe AKI" (n = 59). The VDI did not differ between groups of AKI. After adjustment, "mild and severe AKI" were an independent risk factor for mortality (HR 2.42 95%CI[1.01-5.83], p = 0.048 and HR 1.99 95%CI[1.30-3.03], p = 0.001 respectively). "Severe AKI" had higher levels of plasma IL-10, MIF and IL-6 compared to “no AKI” and mild AKI (p<0.05 for each), with no difference in mHLA-DR at day 0. HLA-DRB genotyping showed a significantly lower proportion of 4 HLA-DRB alleles among patients requiring renal replacement therapy (RRT) (58%) than in patients with severe AKI who did not receive RRT (84%) (p = 0.004).

**Conclusions:**

AKI severity is independently associated with mortality and plasma IL-10, MIF or IL-6 levels. Presence of 4 alleles of HLA-DRB in severe AKI patients seems associated with a lower need of RRT.

## Introduction

Acute kidney injury (AKI) is common in intensive care patients and associated with a worse prognosis [1], [2], [3]. Relatively few studies have reported the potential difference between outcome association and AKI severity grading in severe sepsis or septic shock. The association between AKI and outcome is still poorly understood and is considered related to hypoperfusion [4], [5]. Several arguments challenge this view: not all septic shock patients develop AKI despite similar hypotension and hemodynamic resuscitation; fresh human renal biopsies after death fail to show important ischemic lesions; biopsies show frequent microvessel thrombosis, infiltration by immune cells, and apoptosis [6]; the recently introduced biomarker neutrophil gelatinase-associated lipocalin (NGAL) a metallo-protein from neutrophils, is a good predictor of severe AKI [7] with a large lipocalin gene expression during post-ischemic reperfusion [8]. All of these observations suggest an important role of “renal immune toxicity.” Among these non hemodynamic factors, the intensity of the systemic inflammatory response and genetic factors are reasonable candidates during severe sepsis. In this multicentric study, we sequentially looked at the level of plasma MIF and IL-6 as pro- and IL-10 as anti-inflammatory cytokine to assess inflammatory response. In addition to cytokine plasma levels, monocyte human leukocyte antigen-DR (mHLA-DR) expression was measured as a cellular innate functional test. The focus on major histocompatibility complex (MHC) class II expression came from the well demonstrated rapid (hours) and deep downregulation of HLA-DR in circulating monocytes during severe sepsis [9], [10], [11]. In addition, MHC class II expression protein especially HLA-DR expression is constitutive to peritubular and glomerular capillary endothelium [12], [13]. In acute inflammation, such as acute renal rejection or glomerulonephritis [13], HLA class II protein expression was induced on proximal tubular cells [12] or after stimulation by interferon γ [14]. The selective expression of HLA-DR in kidney microvasculature indicates an unusual regulation of class II that may contribute to the kidney response to foreign antigenes. The HLA-DRB genes, highly polymorphic genes of MHC class II, were chosen to study the HLA-DR genotype. Because these genes are associated together in linkage desequilibrium, we expected this approach would detect a haplotype-phenotype association with greater probability.

## Materials and Methods

### Ethics

This multi-centre study was approved by the AP-HP Cochin Hospital Ethics Committee (# CCPPRB 2061), which was valid for all AP-HP hospitals. Written informed consent was obtained for each patient from the patient himself or next of keen.

### Patients

**Figure 1:**
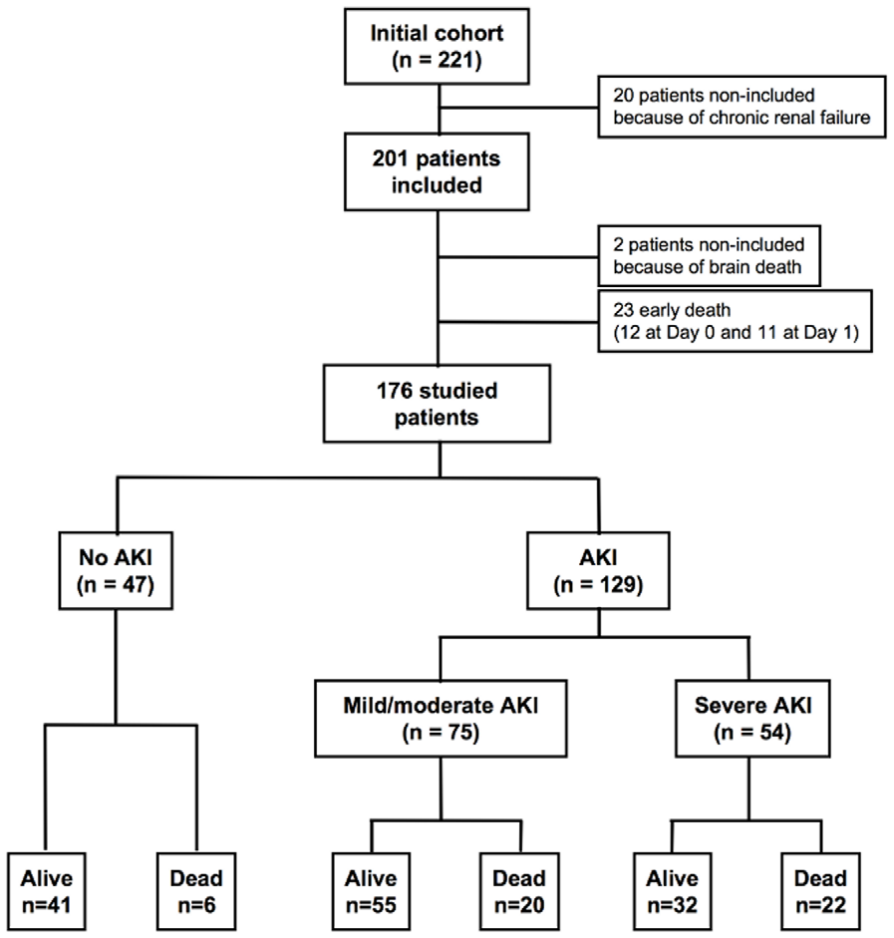
Flowchart of the enrolled septic patients with different AKI severity and outcome.

This study involved patients from 4 Intensive Care Units in Paris, France, including 2 medical and 2 surgical ICUs. Patients fulfilling the criteria of severe sepsis or septic shock defined according to the ACCP/SCCM consensus conference [15] and having at least two organ failures defined by the SOFA (sequential organ failure assessment) score (value >1 for each organ failure were considered) [16] were included between January 2004 and December 2005. Patients with chronic renal failure were excluded (Figure 1). Epidemiological characteristics and common past medical events were collected. Use of vasopressors, inotropes, hydrocortisone, activated protein C, insulin, fluid loading, and renal replacement therapy (RRT) were recorded. Mortality was assessed at the 28^th^ day after enrolment. Patients who died during the first 48 hrs were excluded from this study, considering the time-evolution too short to meet the criteria for AKI definition (Figure 1) [17].

Since the present study was designed before the publication of the RIFLE and AKIN scores [17], [18], patients were classified on the basis of recalculated AKIN score [16] [19]. The concordance between the SOFA_kidney_ and AKIN score classifications was good (91% of patients were identically classified) (Table S1). We report only the results based on AKIN score. The worst value of these renal classifications within the first forty-eight hours (day 2) was recorded. Since the AKIN score is based on creatinine increases from baseline and, in most cases the baseline creatinine level was unknown, it was calculated with the Modified Diet in Renal Disease formula based on a creatinine clearance of 75 ml/min [18]. Patients were classified in three groups: 1) patients without early AKI (serum creatinine≤110 µmol/L; UO >0.5 ml/kg/hr i.e. AKIN 0), 2) patients with mild AKI (AKIN 1 or 2) and 3) patients with severe AKI (AKIN 3). According to the original recommendation [18] for RRT, individuals who receive RRT were considered to have met the criteria for stage 3. Organ failures were classified accordingly to their SOFA score. An organ was considered failing when SOFA score for this organ was of 1 or over 1. To assess the role of shock in the development of early AKI, we stratified patients according to cardiovascular failure using the vasopressor dependency index (VDI) which takes into consideration the vasopressor dose and the mean arterial pressure (MAP). The vasopressor dependency index was calculated as the ratio of catecholamine index to the MAP [20], the catecholamine index being calculated as follows: (dopamine dose×1)+(dobutamine dose×1)+(adrenaline dose×100)+(noradrenaline dose×100)+(phenylephrine dose×100), with all doses expressed as µg/kg/min.

### Description of Procedures or Investigations Undertaken

#### Inflammatory parameters

Blood samples were collected at day 0, day 1, day 2 for measurements of monocyte HLA-DR expression and plasma cytokines.

Monocyte HLA-DR expression was measured by flow cytometry with a FACScan instrument (Becton Dickinson, San Jose, CA) and Cellquest software (Becton Dickinson). Double color staining was performed on whole blood with isotype control antibodies (Simultest control), anti CD14 antibodies attached to fluorescein isothiocyanate (clone MφP9), and anti HLA-DR antibodies attached to phycoerythrin (clone L243), according to the manufacturer recommendations (Becton Dickinson). The anti HLA-DR antibody was raised against the α-subunit of the HLA-DR receptor [21]. The mean fluorescence intensity displayed by anti HLA-DR attached to phycoerythrin was analyzed in the monocyte population, gated as a cell population with appropriate light scattering properties and with CD14 expression. Expression of HLA-DR was quantified by the number of antibodies bound per cell (AB/C) (Quantibrite®, Becton Dickinson) converting the classic mean fluorescence intensity (MFI) after calibration to compare the results obtained with different flowcytometers. The regression slope of correlation between MFI and the estimated AB/C never varied over time and ranged between 0.999 and 1.

Plasma IL-10 and MIF levels were determined by an immunoenzymatic method (ELISA): plasma IL-10 using a PharMingen kit (optEIA™ set, BD PharMingen, San Diego, CA) according to the manufacturer’s recommendations. Plasma MIF was measured using monoclonal anti-human MIF and biotinylated anti-human MIF antibodies, with recombinant human MIF (R&D systems, Abingdon, Oxon, UK). IL-6 plasma levels were measured by Electro-chemiluminescence immunoassay (ECLIA) (Roche Diagnostics) using automated Cobas e601 (Roche Diagnostics).

Standard samples ranged from 7.8 to 500 pg/ml for IL-10 and from 31.25 to 2000 pg/ml for MIF. Detection thresholds were 3.52±3.39 pg/ml for IL-10, 13.36±6.02 pg/ml for MIF and 1.5 pg/ml for IL-6. Normal values in the laboratory were 0.71±0.52 pg/ml for plasma IL-10, 404.28±681.60 pg/ml for MIF and below 7 pg/ml for IL-6.

#### HLA-DRB1 genotyping

The heterodimeric protein receptor HLA-DR [22] consists in one α- and one β-chain, respectively encoded by one α-gene and four functional β-genes, called HLA-DRB1, -DRB3, -DRB4, and -DRB5. HLA-DRB1 is constitutively always present with more than 100 different alleles. The individual HLA-DRB haplotypes correspond to the presence or not of specific combination between HLA-DRB1 gene and one other DRB genes on each chromosome 6. The genotyping included the detection of the HLA-DRB1 alleles, which allowed to define the different haplotypes [23]. The genotypes HLA-DRB3, -DRB4, and -DRB5 were then deducted from their specific associations (linkage dsequilibrium) with the HLA-DRB1 alleles: presence of HLA-DRB3 is linked to the following HLA-DRBB1 alleles: HLA-DRB1*03, DRB1*11, DRB1*12, DRB1*13, or DRB1*14; HLA-DRB4 is linked to: HLA-DRB1*04, DRB1*07, or DRB1*09; and HLA-DRB5 is linked to HLA-DRB1*15 or DRB1*16. [23]. Combination of HLA-DRB genes in an individual genome varies from two genes (one HLA-DRB1 genes on each chromosome 6) to four genes, when the two HLA-DRB1 genes are in linkage disequilibrium with a second HLA-DRB gene. Homozygosity was defined when in addition to both HLA-DRB1 genes, the linkage disequilibrium was with HLA-DRB3 (B3/B3), HLA-DRB4 (B4/B4), or HLA-DRB5 (B5/B5). For HLA-DRB1 typing, the medium resolution genotyping was performed using polymerase chain reaction (PCR) sequence using a specific oligonucleotide reverse dot blot kits (InnoLipa HLA-DRB1 kit, Innogenetics, Zwijndrecht, Belgium).

### Statistical Analysis

Results are expressed as mean (SD) or median (25^th^ to 75^th^ percentiles) while categorical variables were expressed as count and percentage. Comparisons among the three groups (i.e. no AKI, mild AKI and severe AKI respectively) were performed with Kruskal-Wallis test and Chi-square test for continuous and categorical variables respectively. Patients with no AKI and mild AKI were pooled and compared to patients with severe AKI using the Mann-Whitney for continuous variables and Chi-square tests for categorical variables. Cumulative incidence of all-cause in-hospital mortality over time was estimated, considering being alive at discharge as a competing event. The length of stay was computed from 48 hours after ICU admission till the date of death, discharge or transfer. The association of AKI with survival was estimated without and with adjustment on prognostics covariates (namely age, gender, comorbidities (diabetes and cancer) and the SOFA score with exclusion of the renal item, SOFA-II). Logistic regression was used to determine a set of variables independently associated with severe AKI. Variables included in the model were chosen based on their clinical relevance among variables associated with severe AKI at a 0.05 level. The model selection used was a stepwise backward-forward procedure. Discriminative ability of the final model was evaluated by the c- index (identical to the area under the receiver operating characteristics [ROC] curve). Two-way ANOVA analysis for repeated measurement was used to compare variation of different biological values between severe AKI and no or mild AKI and between the two types of genotypes (two or three versus four alleles, see below for details). p<0.05 was considered as statistically significant. All analyses were performed using R 2.6.2 statistical software (The R Foundation for Statistical Computing, Vienna, Austria).

#### Genetic analysis

Allelic frequencies of HLA alleles were calculated by gene counting, and compared between groups either by chi-square test (with Yates correction if needed) or Fisher exact test using 2×2 contingency tables. The significance was set to pc <0.05. Healthy Caucasians (n = 172) constituted the reference population in the lab, HAYEM reference panel [24].

## Results

### Patients

The study flow diagram is shown in Figure 1. Of the 221 screened patients, 20 were not included because of chronic renal failure and 23 additional patients were excluded because of early death (within 48 hours) before the criteria of early AKI could be met. Two additional patients were not included because of brain death. The majority of patients were caucasian (94%) and relatively few patients fit with extreme weights (median weight 71 kg, IQ (60–82)). Only 2 patients free of AKI after 48 hrs later developed AKI. Age and gender did not differ between patients with and without AKI (Table 1). Patients with AKI were less likely to have COPD or chronic respiratory insufficiency compared to patients with no AKI. Occurrence of other organ failure did not differ between groups on inclusion. However, patients with severe AKI had higher severity of illness on admission (for both SAPS II and SOFA scores) compared to the no AKI and mild AKI groups. This was also observed for mild AKI compared to no AKI (Table 1). The site of infection was more frequently the lung in no AKI group than in others (Table 2). Pathogens responsible for sepsis did not differ between groups (Table 2).

**Table 1:** Clinical characteristics of patients at inclusion.

	No AKI (n = 47)	Mild AKI (n = 75)	Severe AKI (n = 54)	p	p1	p2	p3
Age, years	60.1 (15.1)	61 (15.7)	63.3 (15.5)	0.61			
Sexe – male(%)	30 (63.8)	47 (63.5)	34 (63)	1			
Comorbidities, n(%)							
- cardio-vascular	22 (46.8)	30 (40.5)	26 (48.1)	0.69			
- respiratory	9 (19.1)	6 (8.1)	2 (3.7)	0.032	0.092	0.022	0.47
- Diabete	4 (8.5)	16 (21.6)	8 (14.8)	0.16			
- Liver cirrhosis	0 (0)	4 (5.4)	3 (5.6)	0.29			
- Cancer	4 (8.5)	15 (20.3)	10 (18.5)	0.23			
- Immnosuppression	7 (14.9)	4 (5.4)	4 (7.4)	0.21			
Surgical sepsis, n(%)	8 (17)	20 (27)	12 (22.2)	0.47			
Secondary infection, n(%)	14 (29.8)	26 (35.1)	13 (24.1)	0.4			
SOFA score	5.4 (2.1)	7.3 (2.8)	10 (3)	<0.0001	<0.0001	<0.0001	<0.0001
SAPS II	37.8 (12.4)	44.2 (13)	55.3 (13.7)	<0.0001	0.0073	<0.0001	<0.0001
Organ failures	2.6 (0.6)	3.1 (1)	3.6 (1.1)	<0.0001	0.0022	<0.0001	0.038
Organ failures, n(%)							
- Cardio-vascular	43 (91.5)	67 (90.5)	49 (90.7)	1			
- Hematologic	17 (36.2)	26 (35.1)	24 (44.4)	0.53			
- Neurologic	14 (29.8)	22 (29.7)	17 (31.5)	0.98			
- Hepatic	4 (8.5)	13 (17.6)	12 (22.2)	0.17			
- Pulmonary	32 (68.1)	40 (54.1)	39 (72.2)	0.083			

p; comparison between the 3 groups, no AKI, mild AKI and severe AKI. When significant: p1, comparison between no AKI and mild AKI; p2, between no AKI and severe AKI; p3, between mild AKI and severe AKI with a Mann-Whitney –test or Chi2-test. Results are expressed in median (IQR).

**Table 2:** Description of initial infection.

	No AKI (n = 47)	Mild AKI (n = 75)	Severe AKI (n = 54)	p	p1	p2	p3
**Origin of infection n(%)**							
- Pneumonia	34 (72.3)	38 (51.4)	28 (51.9)	0.047	0.024	0.042	1
- Abdomen	8 (17)	15 (20.3)	13 (24.1)	0.72			
- Central nervous system	2 (4.3)	2 (2.7)	1 (1.9)	0.73			
- Urinary tract	0 (0)	10 (13.6)	6 (10.2)	0.29			
- Skin and soft tissues	1 (2.1)	4 (5.4)	6 (11.1)	0.2			
- Blood stream infection	2 (4.3)	6 (8.1)	0 (0)	0.06			
**Pathogens, n(%)**							
-Gram-negative bacteria	9 (26.5)	22 (37.9)	12 (33.3)	0.55			
- Gram-positive bacteria	1 (2.9)	0 (0)	0 (0)	0.27			
- Polymicrobial sepsis	13 (38.2)	16 (27.6)	14 (38.9)	0.42			
- Others	2 (5.9)	2 (3.4)	1 (2.8)	0.72			

p; comparison between the 3 groups, no AKI, mild AKI and severe AKI. When significant: p1, comparison between no AKI and mild AKI, p2, between no AKI and severe AKI, p3, between mild AKI and severe AKI with a Mann-Whitney –test or Chi2-test. Results are expressed in median (IQR).

During the first 48 hours, 30 patients had severe sepsis and 146 were in shock and 36 patients (28% of AKI patients) necessitated renal replacement therapy (RRT) within the first week. The severity of cardiovascular failure expressed as the VDI did not significantly differ between sub-groups of AKI (p value of Kruskal-Wallis 0.63) (Table S2). Patients with severe AKI were more likely to receive hydrocortisone (81.5%) in comparison with mild AKI and no AKI (vs. 51% and 51% respectively) (p<0.00059, Table 3). Patients with severe AKI also received recombinant activated protein C (APC) more often than did patients with mild AKI (18.5% vs. 4.1%) (p<0.00063, Table 3).

**Table 3:** Clinical and biological characteristics of patients over the first 48 hours after inclusion.

	No AKI (n = 47)	Mild AKI (n = 75)	Severe AKI (n = 54)	p	p1	p2	p3
Highest SOFA score	6 (2.3)	8.1 (2.8)	11.6 (3)	<0.0001	<0.0001	<0.0001	<0.0001
Lowest MAP, mmHg	58.5 (12.4)	55.7 (11.8)	44.4 (18.3)	0.00011	0.27	0.00011	0.00053
Mechanical ventilation, n(%)	36 (76.6)	47 (63.5)	46 (85.2)	0.02	0.16	0.31	0.0087
Lowest PaO_2_/FiO_2_ ratio	292 (118)	319(135.6)	277 (150.4)	0.083			
Highest temperature, °C	38.5 (0.8)	38.7 (1)	38.5 (1.1)	0.2			
Highest leukocyte,/mm^3^	17.5 (10.7)	20 (12)	18.6 (12)	0.36			
Lowest leukocyte,/mm^3^	13.9 (8.6)	15.9 (10.6)	14.4 (10.1)	0.38			
Highest glycemia, mmol/l	12.5 (4.3)	13.1 (6.1)	13.4 (5.2)	0.76			
Lowest glycemia, mmol/l	7.1 (2.9)	7.6 (3)	7.5 (3.1)	0.25			
Fluid balance ml	2183 (2497)	3023 (2620)	4368 (2653)	<0.0001	0.031	<0.0001	0.0016
Received Colloids, n(%)	30 (63.8)	58 (78.4)	46 (86.8)	0.024	0.096	0.0097	0.25
Lowest platelet count, 10^3^/mm^3^	234 (144.4)	188 (117.4)	164 (115.3)	0.02	0.13	0.0038	0.17
Lowest prothrombin time, %	75.5 (22.2)	70.2 (18.9)	61.9 (22.7)	0.0047	0.097	0.0029	0.024
Highest bilirubin, mmol/l	1 (1.1)	1.7 (2.5)	2.1 (1.9)	0.0015	0.024	0.00044	0.074
Highest lactate, mmol/l	3.2 (3.2)	3.9 (2.3)	7.3 (6.1)	<0.0001	0.0024	<0.0001	0.0042
Norepinephrine, n(%)	27 (57.4)	50 (67.6)	47 (87)	0.0026	0.33	0.0013	0.012
Dobutamine, n(%)	8 (17)	13 (17.6)	20 (37)	0.021	1	0.028	0.015
Epinephrine, n(%)	9 (19.1)	13 (17.6)	18 (33.3)	0.1			
Dopamine, n(%)	3 (6.4)	6 (8.1)	5 (9.3)	0.94			
HC, n(%)	24 (51.1)	38 (51.4)	44 (81.5)	0.00059	1	0.0014	0.0007
APC, n(%)	0 (0)	3 (4.1)	10 (18.5)	0.00063	0.28	0.0015	0.015
Insulin, n(%)	33 (70.2)	53 (71.6)	36 (72)	1			

p; comparison between the 3 groups, no AKI, mild AKI and severe AKI. When significant p1, comparison between no AKI and mild AKI, p2, between no AKI and severe AKI, p3, between mild AKI and severe AKI with a Mann-Whitney –test or Chi2-test. MAP: mean arterial pressure. PT: prothombin time. Hydrocortisone: HC, activated protein C: APC. Results are expressed in median (IQR).

### Relation between AKI Severity and Outcome

Over the first 48 hours, patients with mild and severe AKI had more frequent non-renal organ failures than "no AKI" group as illustrated by higher SAPSII (p = 0.0073 and p<0.0001 respectively) and SOFA (p<0.0001 for both) scores. “Severe AKI” patients showed similar differences when compared with "mild AKI" for both scores (p<0.0001). Among organ failure items, "severe AKI" had higher serum bilirubin values than "No AKI" (p = 0.00044) and "mild AKI" (p = 0.074), a lower prothrombin values than "no AKI" (p = 0.0029) and "mild AKI" (p = 0.0024), a lower platelet count than "no AKI" (p = 0.0038) and a lower mean arterial pressure than "no AKI" (p = 0.00011) and "mild AKI (p = 0.00053) (Tables 1 and 3). The delay between the 1^st^ and the 2^nd^ organ failure averaged 18 hrs and AKI was the first OF in 75 patients (43%) preceding cardiovascular failure. Two patients without AKI later developed severe AKI and 4 mild AKI patients later developed severe AKI.

**Figure 2:**
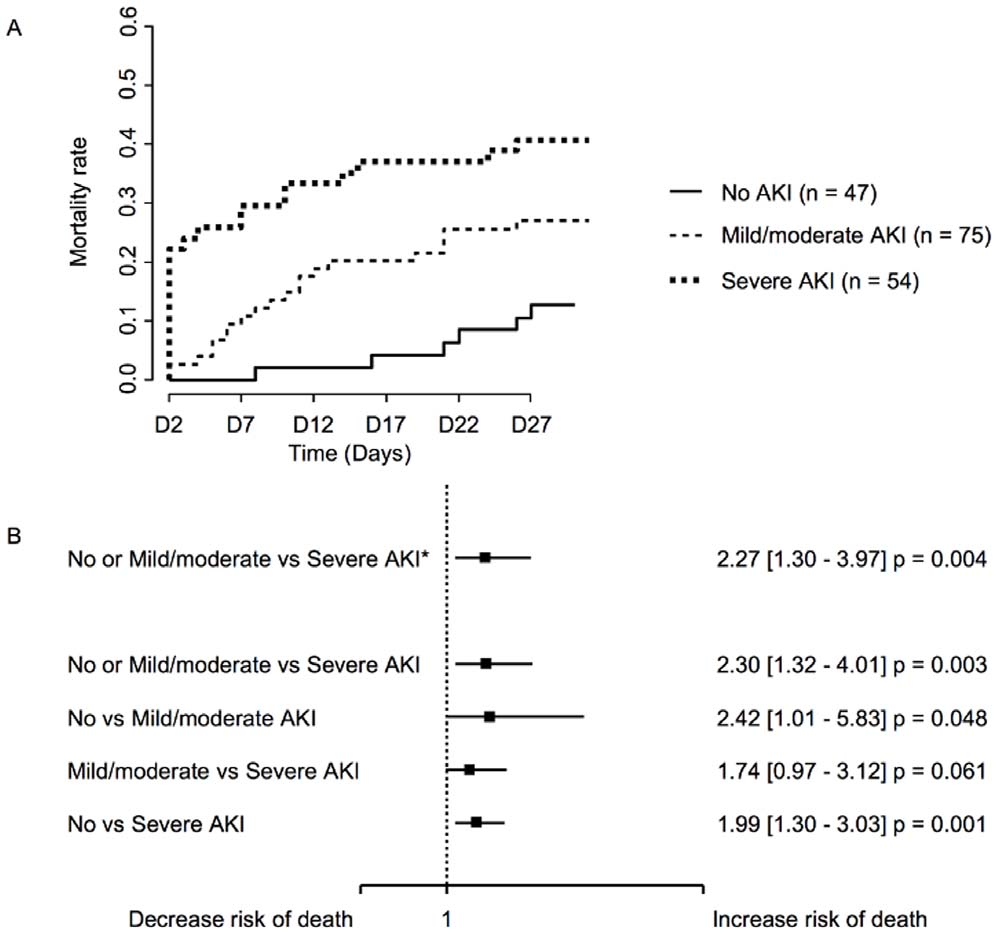
Analysis of mortality according to AKI severity. A. Mortality curves according to AKI severity from day 2 to day 28. **B.** Univariate and multivariate analysis of the association between mortality and AKI. (*) indicates results using competitive risk regression models (analyses adjusted on SOFA-II, age, sex, diabetes and cancer) of mortality risk.

Patients with "severe AKI" had a significantly higher mortality rate at day 28 (40.7%) compared to patients with mild AKI (26%) or no AKI (12%) (p = 0.001) (Figure 2a). The multivariate analysis (Figure 2b) using competitive risks model as an independent factor of mortality after adjusting for age, gender, presence of diabetes and cancer and the SOFA score, excluding the renal item showed differences for "severe AKI" versus "no AKI" or "mild" AKI (Hazard Ratio (HR) 2.27 CI 1.30-3.97; p = 0.004). In absence of adjustment, "mild AKI" versus no AKI (HR 2.42 CI 1.01-5.83; p = 0.048) were significant and "severe AKI" versus "mild AKI" was closed to the significance (HR 1.74 CI 0.97-3.12; p = 0.061).

The logistic regression exploring associated factors with both "mild" and "severe" AKI included the following variables: SOFA score, number of organ dysfunctions, mechanical ventilation, fluid balance, PT, lactate, norepinephrine infusion, dobutamine infusion. For "mild" AKI, SOFA score at admission (OR [95% CI]  = 1.30 [1.10–1.53] for an increase of 1 unit, p = 0.002) and the number of organ failure at admission (OR [95% CI]  = 1.05 [1.00–1.09] for an increase of 1 organ failure, p = 0.034) were found to be independently associated. For "severe" AKI, only SOFA score at admission (OR [95% CI]  = 1.64 [1.41–1.92] for an increase of 1 unit, p<0.0001) was found to be independently associated.

### Relation between AKI and Inflammatory Parameters (Table 4 and Figure 3)

Compared to patients with "no AKI", "mild AKI" had only IL-6 level that reached the significance (p = 0.02). During the first 48 hrs, the highest levels and the trend in "severe AKI" were significantly different than in "no AKI" for IL-6 (p<0.0001), IL-10 (p<0.0001) and MIF (p = 0.0029). Such a difference was observed between "mild AKI" and "severe AKI" for all the measured cytokines (Table 4). Monocyte surface HLADR expression was low compared to healthy volunteers values [9] and did not differ between groups (mild and severe AKI and no AKI) and overtime (Table 4). Using a logistic regression model to explain the relationship between inflammatory pattern and the occurrence of "severe AKI", IL-10 and MIF were found to be independently associated but not IL-6 and HLA-DR (data not shown).

**Figure 3:**
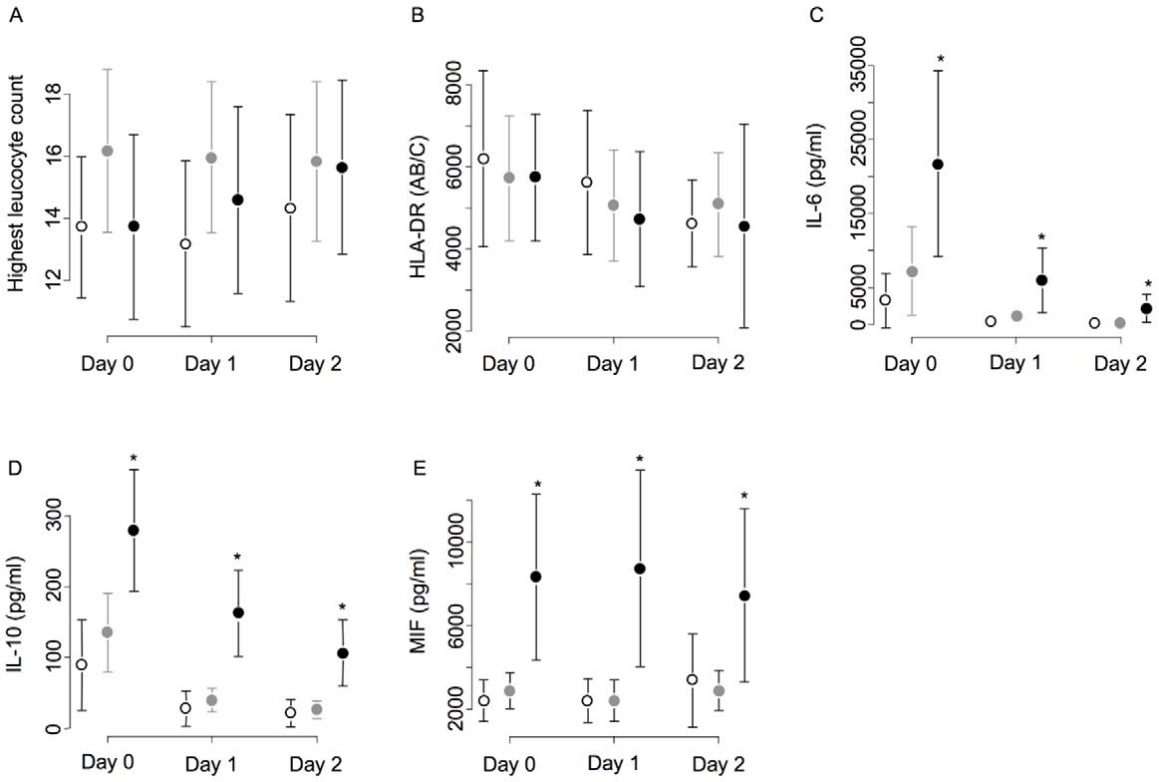
Blood highest leukocytes, monocyte HLA-DR, plasma IL-6, IL-10 and MIF levels in AKI groups. No AKI (white diamond), mild AKI (black diamond) and severe AKI (black circle) at day 0, day1 and day2. Mean and standard deviation are represented.

**Table 4:** Systemic inflammatory parameters (worst values during the 48 first hours).

	patients (n = 176)	No AKI (n = 47)	Mild AKI (n = 75)	Severe AKI (n = 54)	p	p1	p2	p3
HLA-DR	4070	4692	4304	3806	0.57			
(AB/C)	(2640 to 9397)	(2640 to 10769)	(3271 to 84445)	(2205 to 8953)				
IL-10	52.7	24	44.1	141.7	<0.0001	0.052	<0.0001	<0.0001
(pg/ml)	(20. to 202)	(11 to 55.5)	(14.9 to 146.4)	(76.2 to 539)				
MIF	2224	1799	2088	4343	0.0029	0.72	0.0029	0.0031
(pg/ml)	(1033 to 6757)	(755 to 5127)	(786 to 4947)	(1392 to 11989)				
IL-6	491	251	477	3769	<0.0001	0.02	<0.0001	0.002
(pg/ml)	(160 to 38530)	(87 to 532)	(179 to 2489)	(256 to 22823)				

p; comparison between the 3 groups, no AKI, mild AKI and severe AKI. When significant: p1, comparison between no AKI and mild AKI, p2, between no AKI and severe AKI, p3, between mild AKI and severe AKI with a Mann-Whitney –test or Chi2-test. Results are expressed in median (25 to 75th percentile).

### Relation between AKI and HLA-DRB1 Genotype

**Figure 4:**
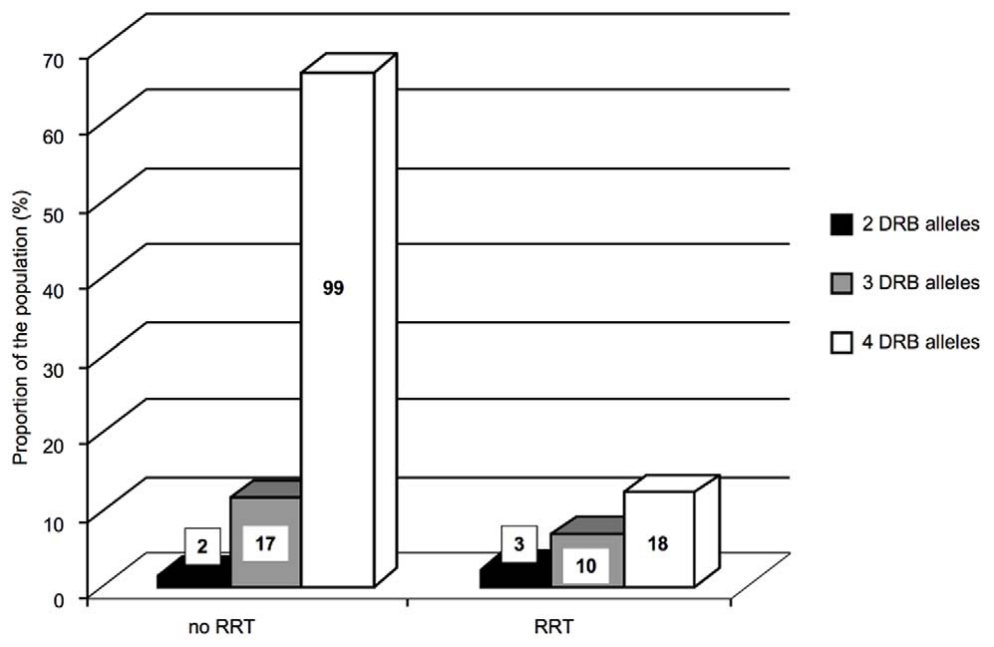
RRT requirement in the population over the first week according to the number of HLA-DRB gene alleles. Black bars in the 2 alleles group, gray bars in the 3 alleles group, white bars in the 4 alleles group.

HLA-DRB1 genotype analysis was performed in 149 patients because of DNA degradation in 26 samples. This DNA degradation concerned 14% of the RRT group and 17% of the remaining patients. The frequencies of HLA-DRB1 alleles and of the pairs of second HLA-DRB alleles are detailed in Tables S3A and S3B. The partition of HLA-DRB1 alleles was similar in this cohort with severe sepsis and septic shock compared to the reference population (Table S3A) [24]. AKI within 48 hours was not associated with a specific HLA-DRB genotype. The genotyping showing only two HLA-DRB1 alleles was more frequent in severe AKI than in the rest of the population ((9%) and (1%) respectively, p = 0.050) (data not shown), but the sample size was too small to draw a definitive conclusion. Considering the presence of three or four alleles (two HLA-DRB1 and two second HLA-DRB genes or two HLA-DRB1 and one second HLA-DRB gene), their incidence did not differ between patients with severe AKI *vs* no or mild AKI. Only in patients presenting four alleles, a trend for a less homozygosity in severe AKI group than in "no AKI" or "mild AKI" was found (p = 0.080). In the RRT population during the 1^st^ week, the incidence of 4 alleles was significantly lower than in the group of patients not treated with RRT (p = 0.004) (Figure 4). In addition, within the 117 patients having 4 alleles, incidence of the homozygotie seemed to be further more protective, with a trend for a lower use of RRT in these patients (8.1%) patients compared to heterozygous (20.6%) (p = 0.070). HLA-DRB genotype was never related to inflammatory phenotypes (Figure S1).

## Discussion

### Statement of Key Findings

The key findings of this study on severe sepsis and septic shock patients are: 1- a graded increase in mortality was associated with the graded AKI severity; 2- severity of AKI did not relate to vasopressor dependency index; 3- plasma cytokine (IL-10, IL-6, and MIF) profiles differed according to AKI severity; 4- support by RRT appeared related to different genotyping profile for HLA-DRB genes, with a “protective" effect of the presence of 4 alleles for HLA-DRB.

### Comparison with Previous Studies

The incidence of AKI observed in this cohort of severe septic patients from medical or surgical origin with a relatively homogenous severity and time evolution for organ failures, is similar to that reported by Bagshaw et al [25]. As previously shown AKI was strongly and independently associated with a poor prognosis, although the mechanism(s) remain unclear. In this study as in previous reports [25], [26], patients having early AKI had higher severity scores and more organ failures compared to those free of AKI. The new finding for severe AKI was the strong association with mortality after application of the competitive risk statistical method in comparison pooling of “no AKI” + “mild AKI.” In addition to its early presence, AKI seemed to remain stable during the study period: only 2 patients started their AKI after the first two days and a low number of patients (n = 4) showed a deteriorating AKI score after 2 days. This suggests that occurrence of AKI and its severity are determined early during the acute phase of severe sepsis.

Our limited understanding of pathogenesis of AKI during severe sepsis gives priority to renal hypoperfusion and ischemia [4], [5]. Recent experimental septic or acute LPS injection inflammatory studies suggest that: renal blood flow follows cardiac output variations, being low with no resuscitation [27] and maintained or high in resuscitated hyperkinetic shock [28]; [29]_;_ renal biopsies performed early after death in septic shock patients failed to observe diffuse and severe ischemic lesion such as renal tubular acute necrosis [6]. In the present study, the VDI [20] did not differ for no AKI, mild AKI and severe AKI groups. In addition, in 43% of the patients, AKI preceded occurrence of shock. Consequently, the classical view of renal hypoperfusion during hemodynamic failure in severe sepsis or septic shock leading to renal ischemia with, acute tubular necrosis and AKI can be questioned and seems to have limited impact. Among the non hemodynamic factors, systemic inflammation might induce a renal “immune toxicity” [30]. The early (the first 2 days) occurrence or not of AKI with few later modifications and the association with initial SOFA score and cytokines suggest such renal “immune toxicity” during the early phase of severe sepsis, when inflammatory processes are most active. Fresh renal tissue biopsies or experimental renal microvessel studies showed acute renal inflammation, with microvessel obstruction [31], prominent tubular cell apoptosis [32] and renal tissue infiltration by immune cells [6], supporting such hypothesis.

### Systemic Inflammatory Phenotype

The role of systemic inflammation in renal injury was nicely shown using a knockout mice model. Mice knock-out for TNF receptors had a better preserved renal function after experimental peritonitis [33]. When a wild type kidney is transplanted into these mice, sepsis induced a severe renal injury only in this transplanted kidney. High plasma IL-10, MIF and IL-6 levels have been associated with severity and a poorer outcome in sepsis [34], [35], [36] and were chosen for the present study. All groups had cytokine levels higher than normal values, which concerned both pro- and anti-inflammatory cytokines. Among the 3 groups, "severe AKI" demonstrated a level of these cytokines higher than in "mild AKI" during the first 48 hrs. The group "mild AKI" was nevertheless higher for some even not all cytokines than patients with no AKI. This group of patients seemed to attenuate inflammation more rapidly, since cytokine levels became not different at day 1 and 2. This suggests a continuum between inflammatory response and severity of AKI. The logistic model confirmed the association between IL-10 and MIF increase and severe AKI. This supports the hypothesis of a renal "immune toxicity" for AKI, especially for the most severe patients. The higher death rate in "mild AKI" compared to "no AKI", despite moderate inflammatory response, suggests a more complex factor interactions than systemic inflammation itself. The type of the measured cytokines did not appear to have specific patterns, although MIF remained higher and stable in "severe AKI". Even previously proposed [37], the mechanistic relation between systemic inflammation and renal injury remains to be demonstrated.

In addition to mediator plasma levels, monocyte human leukocyte antigen-DR (mHLA-DR) expression was measured as a cellular innate functional test. mHLA-DR was rapidly and deeply downregulated in circulating monocytes, but with no prognosis predication as previously shown in severe sepsis and septic shock [9], [10], [38], [39]. This MHC class II expression protein is of importance for kidney injury, since protein HLA-DR expression was described to be constitutive in peritubular and glomerular capillary endothelium in the early 1980s [12], [13]. Kidney HLA-DR protein expression during acute inflammation such as acute rejection and glomerulonephritis [13] showed consistently an induced expression on proximal tubular cells [12]. Similar findings were observed after stimulation by interferon γ [14]. The selective induction of HLA-DR expression in kidney microvasculature indicates an unusual regulation of class II that may contribute to the kidney abilities to respond to foreign antigenes. Considering the different groups of patients in this study (no, mild or severe), there was no significant difference for downregulation of HLA-DR expression in circulating monocytes, but with no information on kidney HLA-DR expression.

### Genotyping of HLA-DRB1

MHC class II and HLA alleles polymorphism have been associated with renal diseases in diabetes type 1 nephropathy [40], [41] or anti-glomerular basement membrane disease [42]. The critiques of these genetic studies focused on single nucleotide polymorphism have been softened by recent Genome wide Association Studies. These studies confirmed the association of HLA alleles with renal diseases as HLA-DQ alpha 1 chain in idiopathic membranous nephropathy [43], HLA-DRB1 and HLA-DQB1 in hepatitis B virus induced nephropathy [44], HLA-DQ and HLA-B in IgA nephropathy [45]. Altogether, these data suggest interactions between MHC class II proteins and glomerular components even when the underlying mechanism remains unknown. In our knowledge, no previous study has reported MHC class II genotyping in severe sepsis and septic shock with AKI severity grading. Although our sample size was relatively small, the result suggests that haplotype of HLA-DRB containing more than two genes (*i.e* three or four) was protective from AKI. The incidence of the haplotype with four genes was significantly lower in patients requiring RRT (severe AKI) than those treated without RRT. Four homozygote HLA-DRB genes seemed even more protective, since the incidence of RRT tended to be less than in heterozygotes. Although the partition of different pattern for HLA-DRB genes did not differ in this septic population from healthy population, these genotyping data identifies a new genetic risk factor for severe AKI. Considering these aspects, homozygosity might be seen as attenuating the initial immune response and the subsequent inflammatory cascade potentially at the kidney tissue level. The present genetic method used for genotyping screened haplotypes and not SNPs only. The differences were present for associated genes having linkage disequilibrium and not for alleles only. This may account for previous failure to show any relation between clinical phenotype and SNPs for this highly polymorphic system.

### Limitations

The findings of this study have to be carefully looked since the sample size was relatively small to draw definitive conclusions. These results have to be confirmed prospectively in a large population. Another limit came from the sepsis timing of evolution, since only the presence of organ failure started to process for enrolment. This implies potential heterogeneity for time evolution between patients and groups. However, the negligible proportion of patients starting AKI after 48 hrs attenuates such limitation.

### Conclusions

This study suggests that AKI severity may reflect the intensity of inflammatory events related to sepsis with particular impact on tissue injury such as the kidney. This signal for inflammation and outcome concern both mild and severe AKI. Inflammatory phenotype and HLA-DRB genotype findings support the concept of inflammation induced severe renal dysfunction with a certain impact of HLA-DRB haplotypes for the most severe AKI requiring RRT. The association between HLA genotype and septic AKI has to be confirmed in larger population, for which Genome Wide Associations method could be applied to evaluate the highly polymorphic MHC class II system and to putative new haplotypes associated with kidney injury.

## Supporting Information

Figure S1 **Inflammatory patterns in relation with HLA-DRB genotype.** Since HLA-DRB haplotypes are different between AKI requiring RRT compared who did not required RRT, we investigated the inflammatory patterns according to haplotypes. No difference was found for monocyte HLA-DR downregulated expression nadir and recovery trend. Similar comparison was made for plasma IL-6, IL-10 and MIF. Blue line corresponded to 4 genes for HLA-DRB; black line corresponded to 2 or 3 genes for HLA-DRB.
(DOC)

Table S1 **Concordance between AKIN and SOFA_kidney_** [**1]**, [2****] **.** 1. de Mendonca A, Vincent JL, Suter PM, Moreno R, Dearden NM, et al. (2000) Acute renal failure in the ICU: risk factors and outcome evaluated by the SOFA score. Intensive Care Med 26: 915–921. 2. Brochard L, Abroug F, Brenner M, Broccard AF, Danner RL, et al. (2010) An Official ATS/ERS/ESICM/SCCM/SRLF Statement: Prevention and Management of Acute Renal Failure in the ICU Patient: an international consensus conference in intensive care medicine. Am J Respir Crit Care Med 181: 1128–1155.
(DOC)

Table S2 **Shows the use of vasopressor and the value of vasopressor dependency index VDI according to kidney injury severity (no, mild, severe AKI) in 146 patients with septic shock** [**3**] **.** The dose of vasoactive/vasopressor agents is expressed as the inotropic score, a dimensionless variable calculated as: (dopamine dose×1)+(dobutamine dose×1)+(adrenaline dose×100) _ +(noradrenaline dose×100)+(phenylephrine dose×100), wherein all doses are expressed as µg/kg/min. 3. Cruz DN, Antonelli M, Fumagalli R, Foltran F, Brienza N, et al. (2009) Early use of polymyxin B hemoperfusion in abdominal septic shock: the EUPHAS randomized controlled trial. Jama 301: 2445–2452.
(DOC)

Table S3 **Shows in part A: the comparison of HLA-DRB1 allele frequencies between severe sepsis and healthy controls; in part B: the comparison of HLA DRB gene frequencies in the severe sepsis and healthy controls.** Interestingly, regarding the second HLA-DRB genes, the B3/B3 genotype was significantly more common in septic patients than in reference population (24% versus 10%, p = 0.01).
(DOC)
