# Supplementary material for: A Multicentre Study of Acute Kidney Injury in Severe Sepsis and Septic Shock: Association with Inflammatory Phenotype and HLA Genotype
Source: PLoS One. 2012 Jun 6;7(6):e35838. doi: 10.1371/journal.pone.0035838 (PMC3368929; doi:10.1371/journal.pone.0035838)
Supplement: Figure S1 — Inflammatory patterns in relation with HLA-DRB genotype. Since HLA-DRB haplotypes are different between AKI requiring RRT compared who did not required RRT, we investigated the inflammatory patterns according to haplotypes. No difference was found for monocyte HLA-DR downregulated expression nadir and recovery trend. Similar comparison was made for plasma IL-6, IL-10 and MIF. Blue line corresponded to 4 genes for HLA-DRB; black line corresponded to 2 or 3 genes for HLA-DRB. (DOC) [file pone.0035838.s001.doc]

**Figure 1S:**
