# Supplementary material for: A Multicentre Study of Acute Kidney Injury in Severe Sepsis and Septic Shock: Association with Inflammatory Phenotype and HLA Genotype
Source: PLoS One. 2012 Jun 6;7(6):e35838. doi: 10.1371/journal.pone.0035838 (PMC3368929; doi:10.1371/journal.pone.0035838)
Supplement: Table S1 — Concordance between AKIN and SOFAkidney [1] , [2] . 1. de Mendonca A, Vincent JL, Suter PM, Moreno R, Dearden NM, et al. (2000) Acute renal failure in the ICU: risk factors and outcome evaluated by the SOFA score. Intensive Care Med 26: 915–921. 2. Brochard L, Abroug F, Brenner M, Broccard AF, Danner RL, et al. (2010) An Official ATS/ERS/ESICM/SCCM/SRLF Statement: Prevention and Management of Acute Renal Failure in the ICU Patient: an international consensus conference in intensive care medicine. Am J Respir Crit Care Med 181: 1128–1155. (DOC) [file pone.0035838.s002.doc]

Table 1S:

|  |  | **AKIN score** | | | |
| --- | --- | --- | --- | --- | --- |
|  |  | **0** | **1** | **2** | **3** |
| **SOFA score** | **0** | **No AKI 41** | 2 | 0 | 0 |
|  | **1** | 6 | **Mild AKI 36** | **0** | 0 |
|  | **2** | 0 | **18** | **12** | 2 |
|  | **3** | 0 | 6 | 1 | **Severe 9** |
|  | **4** | 0 | 1 | 2 | **AKI 40** |
