# Supplementary material for: A Multicentre Study of Acute Kidney Injury in Severe Sepsis and Septic Shock: Association with Inflammatory Phenotype and HLA Genotype
Source: PLoS One. 2012 Jun 6;7(6):e35838. doi: 10.1371/journal.pone.0035838 (PMC3368929; doi:10.1371/journal.pone.0035838)
Supplement: Table S2 — Shows the use of vasopressor and the value of vasopressor dependency index VDI according to kidney injury severity (no, mild, severe AKI) in 146 patients with septic shock [3] . The dose of vasoactive/vasopressor agents is expressed as the inotropic score, a dimensionless variable calculated as: (dopamine dose×1)+(dobutamine dose×1)+(adrenaline dose×100) _ +(noradrenaline dose×100)+(phenylephrine dose×100), wherein all doses are expressed as µg/kg/min. 3. Cruz DN, Antonelli M, Fumagalli R, Foltran F, Brienza N, et al. (2009) Early use of polymyxin B hemoperfusion in abdominal septic shock: the EUPHAS randomized controlled trial. Jama 301: 2445–2452. (DOC) [file pone.0035838.s003.doc]

Table 2S

|  | AKIN score = 0  Or no AKI  (n=47) | AKIN score 1 or 2  Or mild AKI  (n=78) | AKIN score 3  Or severe AKI  (n=51) | P value |
| --- | --- | --- | --- | --- |
| Use of vasopressor | 36 (77) | 63 (81) | 47 (92) | 0.10 |
| Vasopressor dependency index | 0.1 (0 to 0.7) | 0.1 (0 to 1.2) | 0.2 (0 to 2.4) | 0.67 |
