# Supplementary material for: A Multicentre Study of Acute Kidney Injury in Severe Sepsis and Septic Shock: Association with Inflammatory Phenotype and HLA Genotype
Source: PLoS One. 2012 Jun 6;7(6):e35838. doi: 10.1371/journal.pone.0035838 (PMC3368929; doi:10.1371/journal.pone.0035838)
Supplement: Table S3 — Shows in part A: the comparison of HLA-DRB1 allele frequencies between severe sepsis and healthy controls; in part B: the comparison of HLA DRB gene frequencies in the severe sepsis and healthy controls. Interestingly, regarding the second HLA-DRB genes, the B3/B3 genotype was significantly more common in septic patients than in reference population (24% versus 10%, p = 0.01). (DOC) [file pone.0035838.s004.doc]

Table 3SA

| Allele HLA-DRB1*  Frequencies | Septic patients  (n=149) | Healthy controls  (n=172) |
| --- | --- | --- |
| 01 | 0.08 | 0.10 |
| 03 | 0.09 | 0.10 |
| 04 | 0.14 | 0.16 |
| 07 | 0.11 | 0.14 |
| 08 | 0.03 | 0.03 |
| 09 | 0.01 | 0.01 |
| 10 | 0.01 | 0.0 |
| 11 | 0.17 | 0.12 |
| 12 | 0.02 | 0.01 |
| 13 | 0.16 | 0.18 |
| 14 | 0.05 | 0.03 |
| 15 | 0.09 | 0.10 |
| 16 | 0.04 | 0.03 |

Table 3SB :

| DRB gene  Frequencies | Septic patients  (n=149) | Healthy controls  (n=172) |
| --- | --- | --- |
| B3 B3 | 0.24 | 0.10* |
| B3 B4 | 0.28 | 0.23 |
| B3 B5 | 0.11 | 0.10 |
| B3 null | 0.09 | 0.13 |
| B4 B4 | 0.07 | 0.09 |
| B4 B5 | 0.07 | 0.10 |
| B4 null | 0.06 | 0.10 |
| B5 B5 | 0.02 | 0.02 |
| B5 null | 0.03 | 0.02 |
| null null | 0.03 | 0.01 |
